# Accuracy of contrast-enhanced CT in liver neoplasms in children under 2 years age

**DOI:** 10.1007/s00247-024-05958-w

**Published:** 2024-06-03

**Authors:** Aishvarya Shri Rajasimman, Vasundhara Patil, Kunal Bharat Gala, Nitin Shetty, Suyash Kulkarni, Mukta S. Ramadwar, Sajid S. Qureshi, Girish Chinnaswamy, Siddhartha Laskar, Akshay D. Baheti

**Affiliations:** 1 Department of Radiodiagnosis, Tata Memorial Centre & Homi Bhabha National Institute, Mumbai, Maharashtra 400012 India; 2 Department of Pathology, Tata Memorial Centre & Homi Bhabha National Institute, Mumbai, Maharashtra 400012 India; 3 Department of Surgical Oncology, Tata Memorial Centre & Homi Bhabha National Institute, Mumbai, Maharashtra 400012 India; 4 Department of Pediatric Oncology, Tata Memorial Centre & Homi Bhabha National Institute, Mumbai, Maharashtra 400012 India; 5 Department of Radiation Oncology, Tata Memorial Centre & Homi Bhabha National Institute, Mumbai, Maharashtra 400012 India

**Keywords:** Child, Hepatoblastoma, Liver neoplasms, Radiology, Sensitivity and specificity, Tomography

## Abstract

**Background:**

Multiple differentials exist for pediatric liver tumors under 2 years. Accurate imaging diagnosis may obviate the need for tissue sampling in most cases.

**Objective:**

To evaluate the imaging features and diagnostic accuracy of computed tomography (CT) in liver tumors in children under 2 years.

**Methods:**

Eighty-eight children under 2 years with treatment naive liver neoplasms and baseline contrast-enhanced CT were included in this institutional review board approved retrospective study. Two blinded onco-radiologists assessed these tumors in consensus. Findings assessed included enhancement pattern, lobulated appearance, cystic change, calcifications, central scar-like appearance, and metastases. The radiologists classified the lesion as hepatoblastoma, infantile hemangioma, mesenchymal hamartoma, rhabdoid tumor, or indeterminate, first based purely on imaging and then after alpha-fetoprotein (AFP) correlation. Multivariate analysis and methods of comparing means and frequencies were used for statistical analysis wherever applicable. Diagnostic accuracy, sensitivity, and positive predictive values were analyzed.

**Results:**

The mean age of the sample was 11.4 months (95% CI, 10.9–11.8) with 50/88 (57%) boys. The study included 72 hepatoblastomas, 6 hemangiomas, 4 mesenchymal hamartomas, and 6 rhabdoid tumors. Presence of calcifications, multilobular pattern of arterial enhancement, lobulated morphology, and central scar-like appearance was significantly associated with hepatoblastomas (*P*-value < 0.05). Fourteen out of eighty-eight lesions were called indeterminate based on imaging alone; six lesions remained indeterminate after AFP correlation. Pure radiology-based diagnostic accuracy was 81.8% (95% CI, 72.2–89.2%), which increased to 92.1% (95% CI, 84.3–96.7%) (*P*-value > 0.05) after AFP correlation, with one hepatoblastoma misdiagnosed as a rhabdoid tumor. If indeterminate lesions were excluded for biopsy, the accuracy would be 98.8% (95% CI, 93.4–99.9%).

**Conclusion:**

CT had high accuracy for diagnosing liver neoplasms in the under 2-year age population after AFP correlation. Certain imaging features were significantly associated with the diagnosis of hepatoblastoma. A policy of biopsying only indeterminate lesions after CT and AFP correlation would avoid sampling in the majority of patients.

**Graphical Abstract:**

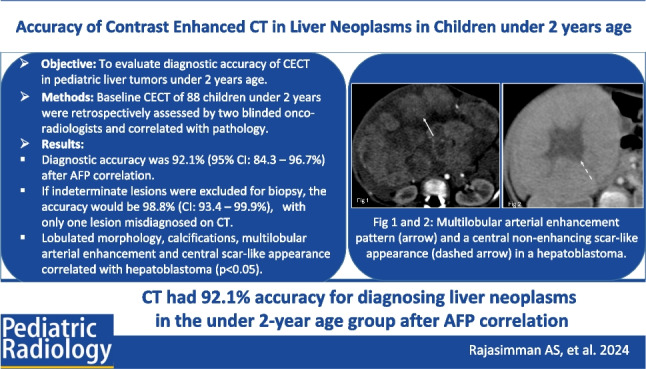

**Supplementary Information:**

The online version contains supplementary material available at 10.1007/s00247-024-05958-w.

## Introduction

Pediatric liver neoplasms comprise of 1–4% of overall pediatric solid tumors [1]. The differential diagnosis for a pediatric liver neoplasm is very different from the adult population, and indeed varies substantially depending on the child’s age. Benign neoplasms predominate in newborns and infants, the commonest being hemangiomas with a prevalence of approximately 4–5% in newborns [2, 3]. The incidence of malignant tumors increases after 6 months of age. Hepatoblastoma dominates the below 5-year population with its peak incidence between 6 months and 3 years [4, 5]. Rare tumors like mesenchymal hamartoma and rhabdoid tumor are also prevalent in under 2-year population with a median age between 11–18 months [3]. Other liver lesions like hepatic adenoma, focal nodular hyperplasia (FNH), hepatocellular carcinoma (HCC), and undifferentiated embryonal sarcomas (UES) are very rare in children under 2 years. These are more common among young and school-going children [3].

The most common clinical presentation is an abdominal swelling with or without pain. Rarely, the patient may present with abdominal distension and vomiting due to tumor rupture [6]. Ultrasound (US) is the first investigation of choice. Once a liver neoplasm is diagnosed on US, further characterization is performed with cross-sectional imaging in the form of computed tomography (CT) or magnetic resonance imaging (MRI). MRI has excellent soft tissue resolution and no radiation exposure. However, the increased scan time, limited availability, and need for sedation have made CT the most commonly utilized modality of investigation in many countries owing to its easy availability and affordability.

Hepatoblastoma is a secretory tumor and demonstrates elevated serum alpha-fetoprotein (AFP) levels. Serum AFP levels may be slightly high during the first few months of life due to the role of AFP in fetal development but do normalize subsequently. AFP level adjusted for age (based on nomogram values) is used as a marker of diagnosis, response assessment, and monitoring. These are either sporadic or associated with Beckwith-Wiedemann syndrome or trisomy 18 [7].

While many of these lesions have classic imaging features, a biopsy under sedation/anesthesia may be required for definitive diagnosis. Recent Pediatric Hepatic International Tumor Trial (PHITT) updates state that a biopsy can be avoided when clinical and imaging findings are concordant in patients with hepatoblastoma or HCC [8]. This strategy may be extended to other pediatric hepatic lesions as well. The objective of this study is to assess the accuracy of contrast-enhanced CT (CECT) in diagnosing pediatric liver tumors in children under 2-years of age.

## Materials and methods

### Patient selection

**Fig. 1:**
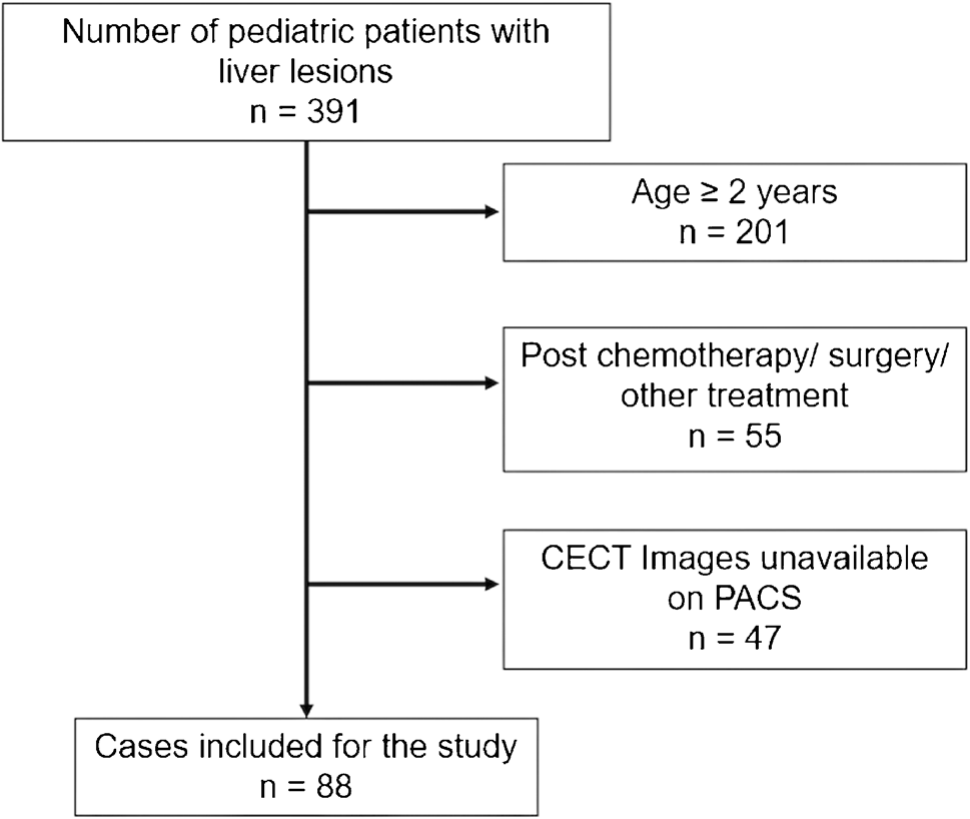
Flowchart illustrating the patient selection process

The study was conducted after the approval from the institutional review board. We performed a retrospective study over the period of 2013 to 2023. Three hundred ninety-one pediatric patients with liver tumors treated in the hospital were retrieved from the institutional pediatric solid tumor group database. Our inclusion criteria were patients who were under 2 years of age at the time of initial presentation, who had baseline imaging available on institutional picture archiving and communication systems (PACS), and who were treatment naive at the time of the study. A total of 88 patients were analyzed after applying the criteria (Fig. 1).

### Clinical and histopathological data

Clinicopathological and follow-up details were retrieved from institutional electronic medical records, including patient demographics, serum AFP levels, treatment provided, and duration of follow-up. Histopathology was confirmed by a dedicated pediatric oncopathologist from our institute. In cases of hemangiomas which were not operated, clinical follow-up and regression on imaging were used for confirmation of diagnosis.

### Imaging and imaging assessment

Scans were performed on a Siemens Somatom 16-slice CT or a 16-slice Siemens Somatom Sensation Open CT (Siemens Healthcare, Erlangen, Germany) using the institutional pediatric liver protocol, a single portal venous phase CT with 1.5 mm slice thickness and axial plane acquisition using intravenous contrast (Omnipaque™ 300, GE healthcare, Marlborough, Massachusetts, USA). Adaptive collimation was used as per the size of the patient’s abdomen. Additional late arterial and delayed phase images were obtained for suspected infantile hemangiomas. Multiplanar reconstructions were made in coronal and sagittal planes. Cases with CECT performed elsewhere but with DICOM images of adequate quality (at least 1.5 mm slice thickness and no artifacts obscuring the lesion) available on PACS were also included. The scans from outside hospital or centers were either dual or triple phase. Finally, 76/88 (86%) cases had late arterial phase, and 69/88 (78%) had delayed phase imaging available as well. The images were reviewed on the Centricity PACS RA1000 (GE HealthCare, Barrington, IL, USA) workstation. A systematic review of all images was performed in consensus by two dedicated pediatric radiologists with 20 years and 15 years of experience respectively.

**Fig. 2:**
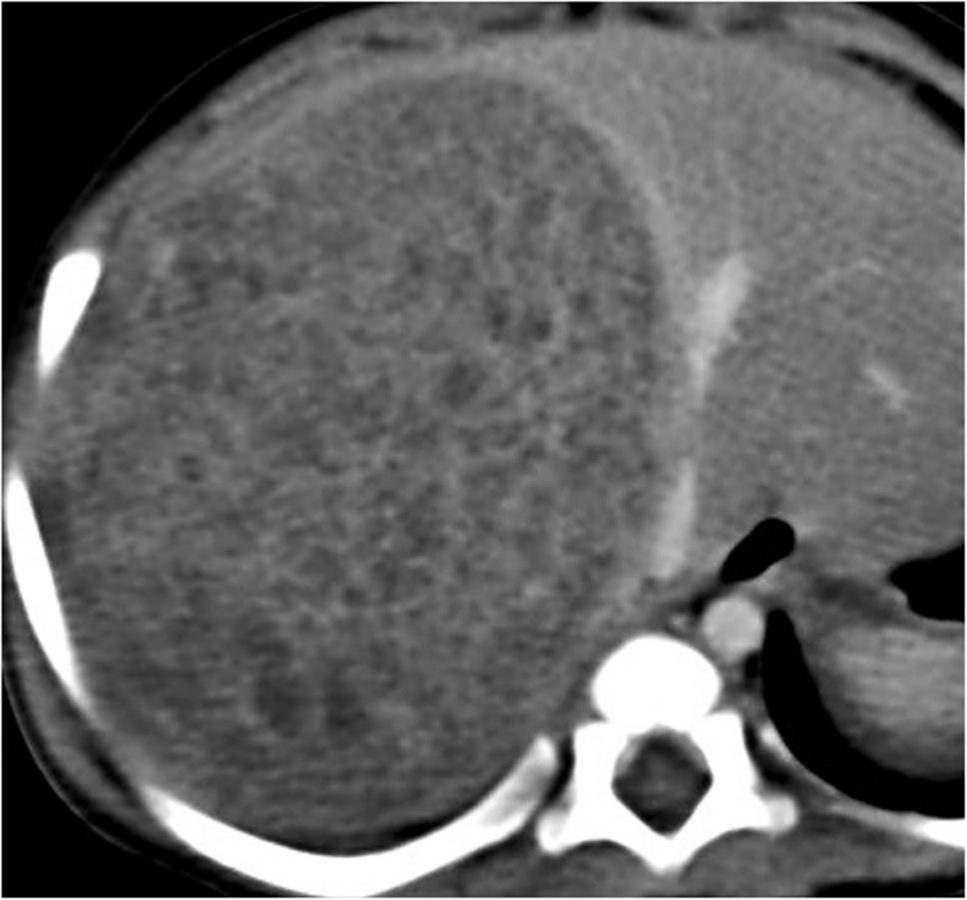
Axial portal venous phase CT abdomen of a 15-month-old boy with mesenchymal hamartoma shows a well-defined lesion with multiple smaller cysts, giving a “sieve-like” appearance

**Fig. 3:**
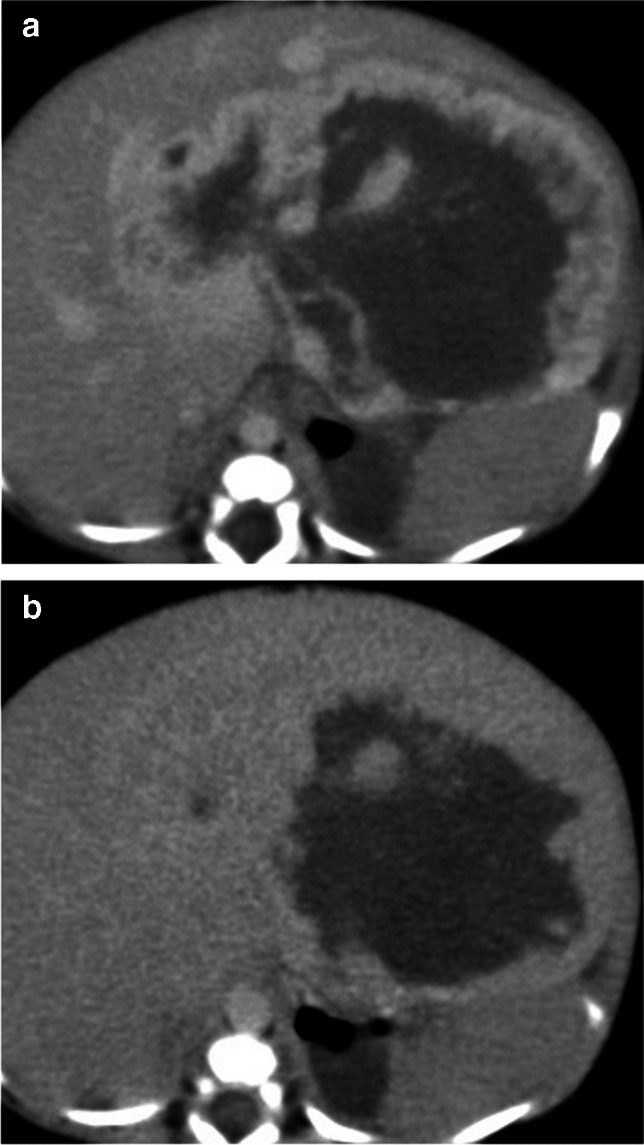
A 10-month-old female presented with abdominal swelling. Late arterial phase (**a**) shows a peripherally enhancing lesion matching the blood pool that shows filling-in on delayed images (**b**)

Imaging features assessed included the size of the lesion, presence of calcifications, cystic change (including the classic “sieve-like” or “Swiss-cheese” multilocular cystic appearance of mesenchymal hamartomas) (Fig. 2), or hemorrhage, and the pattern of enhancement, namely presence of arterial hyperenhancement, washout, and delayed filling-in (in comparison to the rest of the liver parenchyma). The presence of a classic infantile hemangioma pattern, namely peripheral arterial enhancement with centripetal filling-in on delayed phase, was noted (Fig. 3) [9]. The presence of central scar-like appearance was also recorded, defined as a central stellate hypo-enhancing hypo-dense appearance. Other features like enlarged nodes and metastasis were noted as well.

**Fig. 4:**
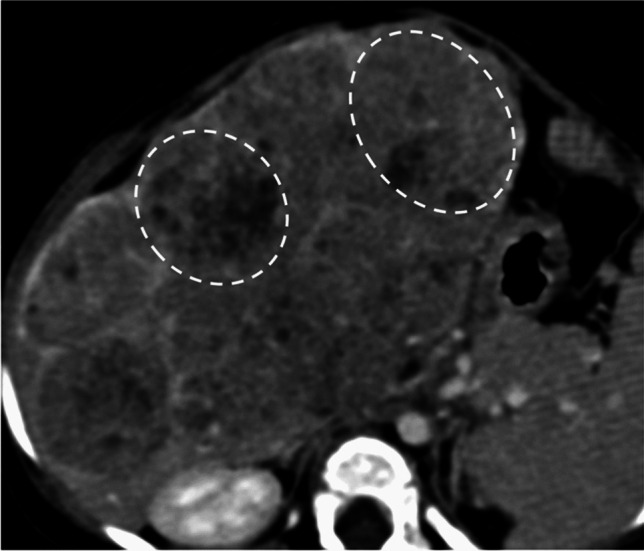
Axial portal venous phase abdominal CT in a 12-month-old girl with hepatoblastoma demonstrating its lobulated morphology (dashed circles)

**Fig. 5:**
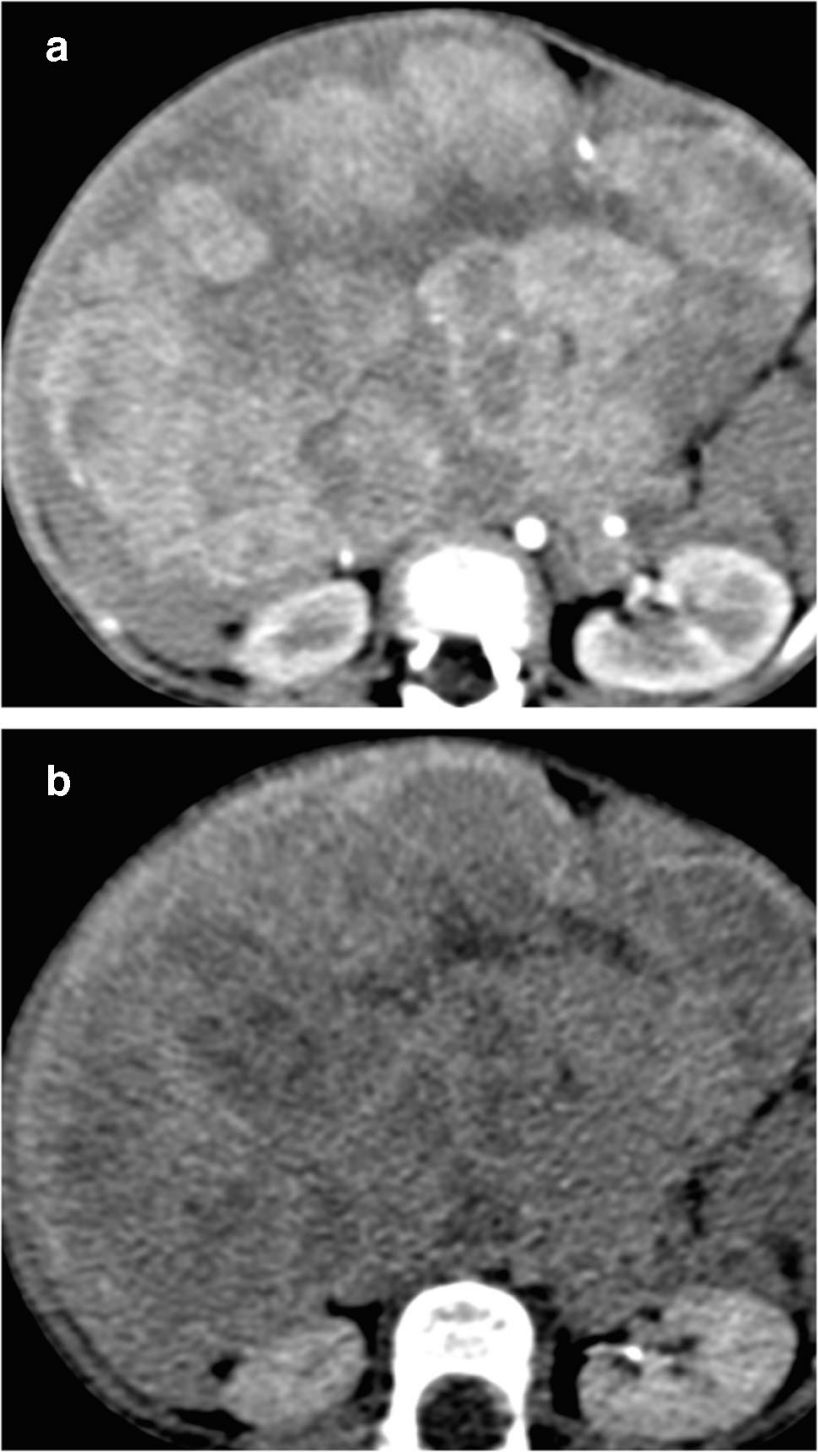
CECT abdomen in a 16-month boy presenting with abdominal lump. **a** Axial arterial phase image showing multilobular pattern of arterial enhancement (arrow) seen as arterially enhancing lobules (dashed circles), consistent with hepatoblastoma. **b** The lobulated morphology is less well appreciated on the portal venous phase, which demonstrates a heterogeneously enhancing hepatic lesion

Both the radiologists were initially blinded with respect to the AFP levels and the final pathology, mimicking the common clinical scenario. They made a diagnosis among “hemangioma,” “hepatoblastoma,” “mesenchymal hamartoma,” or “rhabdoid tumor,” and labeled a lesion “indeterminate and requires biopsy” when in doubt. The diagnosis was based on the lesion’s imaging characteristics, presence of metastases, and patient age. The AFP levels (including age-based cut-offs based on nomogram values) were then provided in the same sitting, and the radiologists were allowed to revise the original diagnosis if they deemed so. Hemangiomas were identified based on their classic enhancement pattern. A multicystic tumor was considered mesenchymal hamartoma. A lobulated heterogeneous solid mass with calcifications or scar-like appearance or presence of multilobular arterial enhancement and raised AFP levels was considered hepatoblastoma. A lesion was labeled lobulated in morphology when it had multiple appreciable lobules within it (Fig. 4). Arterial enhancement within the various lobules of a lesion was deemed multilobular arterial enhancement (Fig. 5). The definitions and illustrations of the imaging characteristics are presented in supplementary Table 4.

Heterogeneous masses without classic features of hepatoblastoma, excessive necrosis, adenopathy, and normal AFP values were labeled rhabdoid tumors. Lesions were labeled indeterminate if the diagnosis remained uncertain due to overlapping or non-specific features.

The sensitivity, specificity, positive and negative predictive value, and the accuracy of imaging in diagnosing hepatic lesions both without and with AFP levels were evaluated. Lesions labeled indeterminate were considered inaccurate for the purposes of analysis. Multivariate analysis of the imaging features vis-à-vis final pathology was performed wherever applicable. Categorical variables were compared using the chi-square test or Fishers’ exact test as appropriate. A *P*-value < 0.05 was considered statistically significant. Statistical analysis was performed using SPSS (the statistical package for social sciences), IBM Corp, released 2012, IBM SPSS Statistics for Windows, Version 26.0. Armonk, NY, IBM Corp.

## Results

The study included 50 boys and 38 girls with a mean age of 11.4 months (95% CI, 10.9–11.8). The mean size of the lesion was 9.7 cm (95% CI, 9.3–10.1) Overall, there were 72 (82%) hepatoblastomas, six infantile hemangiomas (7%), six rhabdoid tumors (7%), and four mesenchymal hamartomas (4%) in the study (Table 1). Hepatoblastomas constituted 77% of the tumors in the under 1-year population (43/56). No patient had a known syndrome or underlying predisposition.

**Table 1:** Demographical and clinical data. *CI* – confidence interval

Variables	*N*	Percent	Mean	95% CI
Age	88		11.4 months	10.9–11.8 months
Gender (male:female)	50:38	57%:43%		
Alpha-fetoprotein levels	88		324,675 ng/mL	129,190–473,604 ng/mL
Pathological diagnosis				
Hepatoblastoma	72	81.8%		
Hemangioma	6	6.8%		
Mesenchymal hamartoma	4	4.5%		
Rhabdoid tumor	6	6.8%		
Size of the lesion			9.7 cm	9.3–10.1 cm

Twenty-nine out of seventy-six (38%) cases demonstrated arterial enhancement, including all hemangiomas (*n*, 6/6) and 23/72 hepatoblastomas (32%). The classic infantile hemangioma pattern of peripheral arterial enhancement and delayed filling-in was observed in 5/6 hemangiomas, with the one remaining case not having delayed phase images available. Two hemangiomas were pathologically proven; the other four hemangiomas had a median follow-up of 1.5 years and showed regression of the lesions on ultrasound.

Twenty-one out of seventy-two (29.2%) hepatoblastomas demonstrated multilobular arterial enhancement (*P*-value = 0.01) (Fig. 4). Lobulated morphology was found in 41/88 (46.6%) cases, of which one was rhabdoid tumor and the rest were hepatoblastomas (*P*-value = 0.01) (Fig. 5).

**Fig. 6:**
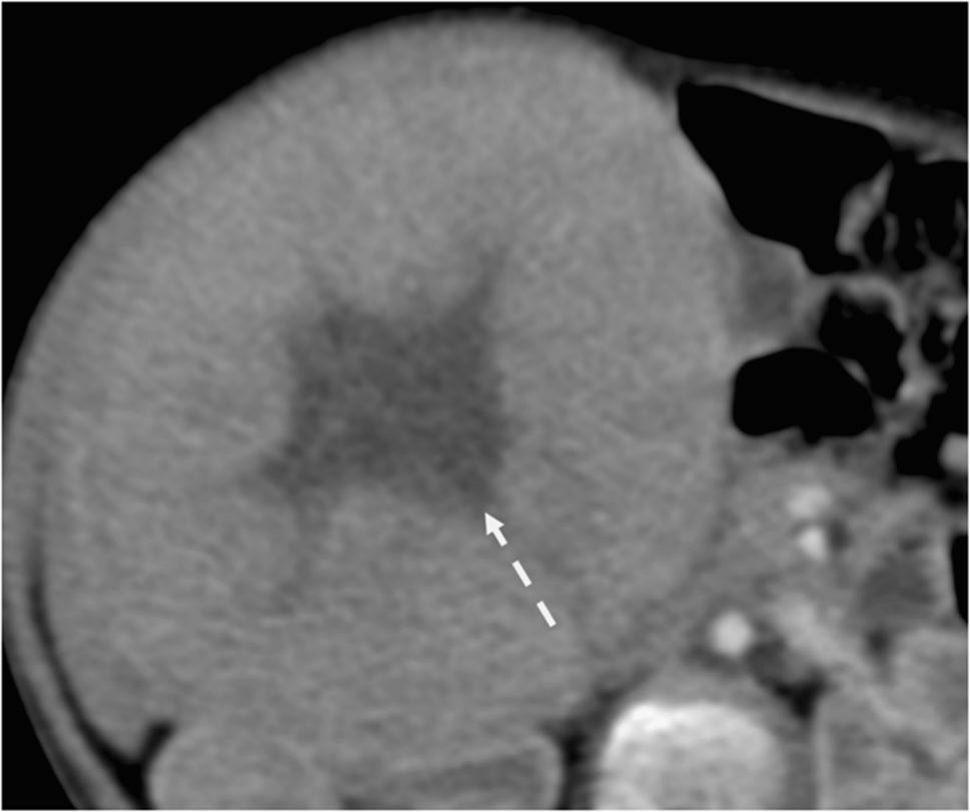
Thirteen-month-old girl with abdominal lump. Axial portal venous phase image shows a lobulated mass with central hypo-enhancing scar-like appearance (arrow), consistent with hepatoblastoma

Eighty-one out of eighty-eight lesions were hypo-enhancing compared to the hepatic parenchyma on the portal venous phase. Five out of eighty-eight lesions were hyperattenuating on the venous phase, all being hemangiomas, while two hepatoblastomas were heterogeneously isoattenuating. Eighteen out of eighty-eight (20%) cases demonstrated a central scar-like appearance (Fig. 6), all of which were hepatoblastomas (*P*-value = 0.01). Twelve out of eighty-eight cases had multifocal lesions, of which 3 were hemangiomas and 9 were hepatoblastomas.

Thirty-three out of eighty-eight (87%) cases demonstrated calcifications, with 30 of them being in hepatoblastomas (*P*-value = 0.04), along with two infantile hemangiomas and one rhabdoid tumor. Twelve out of eighty-eight (14%) cases showed intralesional hemorrhage, with all 12 being hepatoblastomas. No statistically significant association was however present. Further details are tabulated in Table 2.

**Table 2:** Multivariate analysis of clinical and lesion characteristics

Pathologic diagnosis	Hepatoblastoma (*n* = 72)	Hemangioma (*n* = 6)	Mesenchymal hamartoma (*n* = 4)	Rhabdoid tumor (*n* = 6)	*P*-value
Age in months (mean and SD)	11.95 (± 5.1)	5.3 (± 4.5)	15.5 (± 4.2)	7.7 (± 3.2)	** < 0.001**
Sex (M, F)	M = 40, F = 32	M = 2, F = 4	M = 3, F = 1	M = 5, F = 1	0.09
Size in cm (mean and SD)	10.1 (± 2.6)	5.8 (± 2.1)	9.3 (± 3.1)	9.4 (± 0.96)	0.31
Arterial phase enhancement (*n*, 76)	23 (30.3%)	6 (7.9%)	0	0	0.12
Delayed filling-in (*n*, 69)	0	5 (83.3%)	0	0	0.34
Central scar-like appearance	18 (25%)	0	0	0	**0.01**
Calcification	30 (41.7%)	2 (33.3%)	0	1 (16.7%)	**0.04**
Hemorrhage	12 (16.7%)	0	0	0	0.16
Metastasis	11 (15.3%)	0	0	4 (66.7%)	0.34
Multilobular arterial enhancement	21 (29.17%)	0	0	0	**0.013**
Lobulated appearance	40 (55.6%)	0	0	1 (16.7%)	**0.01**

*P*-values in bold format are statistically significant (<0.05)

The median AFP level was 324,675 ng/mL [95% CI, 902,119–1,150,532] and the AFP values were age-matched during interpretation.

Rhabdoid tumors were not diagnosed on isolated imaging alone. After AFP correlation, radiologists identified 3/6 rhabdoid tumors accurately, with a sensitivity of 50% [95% CI, 11.8–88.9%]. Three out of four mesenchymal hamartomas were identified on imaging, with two demonstrating the classic multilocular cystic appearance. No statistically significant imaging features were observed for mesenchymal hamartomas or rhabdoid tumors.

Fifteen out of eighty-eight (17%) cases had metastasis at presentation, including 11 hepatoblastomas and four rhabdoid tumors. Eight cases presented with rupture that includes seven hepatoblastomas and one rhabdoid.

**Fig. 7:**
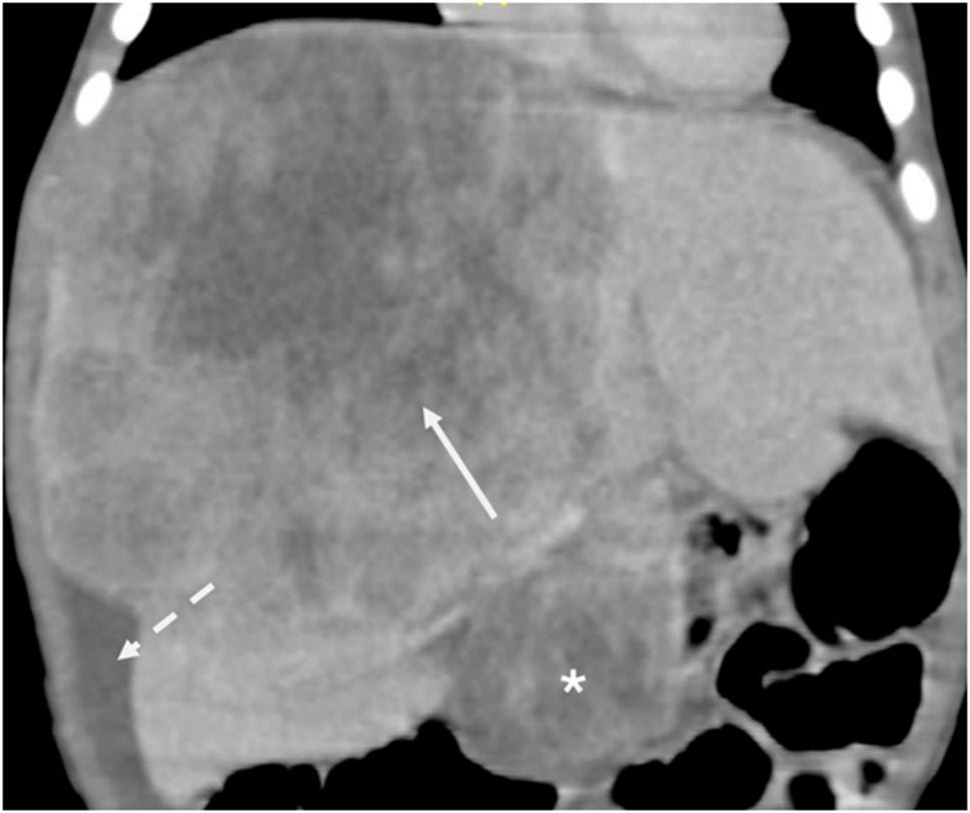
Eleven-month-old girl presenting with irritability and abdominal distention. Coronal CECT image in portal venous phase shows a heterogeneously enhancing hepatic mass (solid arrow) with enlarged partially necrotic locoregional adenopathy (asterisk) and perihepatic fluid secondary to rupture (dashed arrow). This was labeled a rhabdoid tumor due to the adenopathy and normal AFP levels but was proven hepatoblastoma on biopsy

Overall, there were 14 indeterminate lesions without AFP correlation, and six indeterminates after AFP correlation. The six lesions included two hepatoblastoma with borderline AFP levels, a mesenchymal hamartoma with more solid and less cystic components, and three rhabdoid tumors. Apart from the indeterminate lesions, one case of pathologically proven ruptured hepatoblastoma with enlarged nodes and mild ascites with serum AFP of 8 ng/mL was incorrectly labeled as rhabdoid tumor (Fig. 7). Two out of fourteen indeterminate cases had single-phase imaging (one hemangioma and one hepatoblastoma) and the case of hepatoblastoma remained indeterminate after AFP correlation. All the other lesions were correctly classified on imaging.

Assuming the indeterminate lesions as incorrect, the overall accuracy of CECT for diagnosis of pediatric hepatic lesions was 81.8% [95% CI, 72.2–89.2%] and 92.1% [95% CI, 84.3–96.7%] without and with AFP correlation respectively (Table 3). The increase in accuracy after AFP correlation was not statistically significant (*P*-value = 0.118). If a policy of biopsying the indeterminate lesions while avoiding biopsy in the rest was followed, only 6/88 (6.8%) tumors would need sampling. After excluding the indeterminates for biopsy, the accuracy of CT in characterizing the remaining hepatic lesions would be 98.8% (CI, 93.4–99.9%), with overall sensitivity and positive predictive value of 100% (CI, 95–100%) and 98.8% (CI, 98.7–98.8%).

**Table 3:** Evaluation of diagnosis with imaging and after AFP correlation. Note: Cases labeled “indeterminate” were also considered inaccurate

Diagnosis	Accuracy [95% CI]		Sensitivity [95% CI]		Specificity [95% CI]		PPV [95% CI]	
	Before AFP	After AFP	Before AFP	After AFP	Before AFP	After AFP	Before AFP	After AFP
Hepatoblastoma (*n*, 72/88)	**89.8%** [81.5–95.2%] *n*, 79/88	**96.6%** [90.4–99.3%] *n*, 85/88	**90.3%** [80.9–96%] *n*, 65/72	**95.8%** [88.3–99.1%] *n*, 69/72	**87.5%** [61.7–98.5%] *n*, 14/16	**100%** [79.4–100%] *n*, 16/16	**97%** [89.8–99.2%] *n*, 65/67	**100%** [94.5–100%] *n*, 69/69
Hemangioma (*n*, 6/88)	**98.9%** [93.8–99.9%] *n*, 87/88	**100%** [95.9–100%] *n*, 88/88	**83.3%** [35.9–99.6%] *n*, 5/6	**100%** [54.1–100%] *n*, 6/6	**100%** [95.6–100%] *n*, 82/82	**100%** [95.6–100%] *n*, 82/82	**100%** [47.8–100%] *n*, 5/5	**100%** [54–100%] *n*, 6/6
Mesenchymal hamartoma (*n*, 4/88)	97.7%* [92–99.7%] *n*, 86/88	98.9%* [93.8–99.1%] *n*, 87/88	**50%** [7–93.2%] *n*, 2/4	**75%** [19.4–99.3%] *n*, 3/4	100%* [95.7–100%] *n*, 84/84	100%* [95.7–100%] *n*, 84/84	**100%** [16–100%] *n*, 2/2	**100%** [29.2–100%] *n*, 3/3
Rhabdoid tumor (*n*, 6/88)	93.2%* [85.7–97.5%] *n*, 82/88	95.5%* [88.7–98.8%] *n*, 84/88	**0%** *n*, 0/6	**50%** [11.8–88.9%] *n*, 3/6	100%* [95.6–100%] *n*, 82/82	**98.78%** [93.4–99.9%] *n*, 81/82	NA *n*, 0/0	**75%** [26.7–96.1%] *n*, 3/4
Overall CT performance (*n*, 88)	**81.8%** [72.2–89.2%] *n*, 72/88	**92.1%** [84.3–96.7%] *n*, 81/88	**83.7%** [74.2–90.8%] *n*, 72/86	**93.1%** [85.6–97.4%] *n*, 81/87	NA**	NA**	**97.3%** [97–97.5%] *n*, 72/74	**98.8%** [98.7–98.9%] *n*, 81/82
Overall CT performance (excluding indeterminates) (*n*, 82)	**98.8%** [93.4–99.9%] *n*, 81/82		**100%** [95.6–100%] *n*, 81/81		NA**		**98.8%** [98.7–98.8%] *n*, 81/82	

^*^The increase in accuracy and specificity is owing to the increased number of true negatives. **In overall assessment, there are no true negatives to calculate specificity. *AFP* – alpha-fetoprotein, *NA* – not applicable, *PPV*—positive predictive value

## Discussion

CT is a commonly performed investigation for pediatric liver lesions. It is equivalent to MRI in staging the disease, including vascular evaluation [10]. Furthermore, CT chest for screening lung metastasis can also be performed in the same setting [10]. Although there is a potential risk related to radiation exposure on CT, it may get counterbalanced by the need for sedation or anesthesia for MRI [11].

The performance of CT/MRI in lesion resectability in pediatric liver tumors has been extensively studied [10, 12–16]. Though CT is commonly performed, studies assessing the accuracy of CT in diagnosing pediatric liver neoplasms are limited. A retrospective study in 2018 on 41 pediatric liver lesions (< 18-year-old patients) assessed the accuracy of CT and MRI in differentiating benign from malignant lesions based on Liver Reporting and Data System (LI-RADS) criteria. They showed a 100% (CI, 74–100%) sensitivity and 100% (CI, 81–100%) negative predictive value for diagnosing malignant liver lesions [17]. This study however did not diagnose the individual lesion pathology beyond labeling them benign or malignant, was not focused on the < 2 years age group, and encompassed seven hepatoblastomas, three hepatocellular carcinoma, and other predominant benign etiologies. No AFP correlation was performed [17].

Few other studies have evaluated the accuracy of imaging in specific diagnoses. A study evaluating focal liver lesions in children under 14 years in 45 patients found CT/MRI to be more sensitive and specific (95.2% and 90.2% respectively) in diagnosing hemangioma compared to ultrasound, when combined with clinical features like age, size of the lesion, and AFP values, with an AUC value of 0.93 (0.81–0.99) [6]. This is similar to our findings, with the classical enhancement pattern being pathognomonic for the diagnosis. Another 2018 study evaluating the accuracy of CT in diagnosing hepatoblastoma in 43 children (under 18 years age) showed an accuracy of 78.5% for tumors more than 4.5 cm and 71.4% for tumors < 4.5 cm on CT [18]. We observed a higher accuracy of 89.8% for our hepatoblastoma subset of patients on imaging without AFP correlation, which increased to 96.6% with AFP correlation.

There was a significant association between the presence of calcifications and hepatoblastomas, with 42% of hepatoblastomas in our study demonstrating calcifications. More than 50% of hepatoblastomas present with calcifications as per literature, and the commonly described patterns are coarse, chunky, and speckled types [19, 20]. The morphological patterns of calcifications we observed include focal and punctate, coarse/chunky, and curvilinear/rim calcifications. A few rare cases of predominantly calcified hepatoblastomas have also been reported, although we did not observe one in our cohort [21].

A hypo-attenuating central scar has been well established in FNH and fibrolamellar HCC but has also been described previously in hepatoblastoma [22]. Fibrolamellar HCC is not a differential diagnosis in this age group. FNH demonstrates intense homogeneous arterial enhancement and becomes isodense on the portal venous and delayed images, with the scar demonstrating delayed enhancement [23]. This was different from the hypo-enhancing scar-like appearance we observed in 25% of our cases, which significantly correlated with the diagnosis of hepatoblastoma. The pathological correlation for this morphology is not clear and needs further exploration. It could possibly represent central stroma between the tumor lobules. Larger studies are needed to validate this observation.

The diagnosis of rhabdoid on imaging alone was challenging. Aggressive features like metastasis, hemorrhage, necrosis, capsule rupture, or ascites without the typical morphology of hepatoblastoma were labeled indeterminate by the radiologists. The diagnosis was revised to hepatoblastoma or rhabdoid or retained as indeterminate after AFP correlation, given that rhabdoid tumors will not have raised AFP levels. Three cases with predominant cystic/necrotic components and metastases were accurately diagnosed as rhabdoid tumors after AFP levels, while the remaining three remained indeterminate.

PHITT guidelines state that if there are no differential diagnoses other than hepatoblastoma, a biopsy can be avoided after a multidisciplinary team discussion when the imaging and clinical findings are concordant [8]. In cases where the diagnosis is not certain or when a malignant lesion is suspected, the pediatric LI-RADS working group recommends a biopsy [24]. As per the German Society of Pediatric Hematology and Oncology (GPOH) in children below 3 years, a suspected case of hepatoblastoma with highly elevated AFP can be treated as hepatoblastoma without a biopsy [25]. This has been followed in many institutes since children less than 2 years are at higher risk for procedure or sedation/anesthesia related complications. Hemangiomas are also traditionally diagnosed on imaging alone without a biopsy in both children and adults.

With other differential diagnoses of a mesenchymal hamartoma and a rhabdoid tumor also present in the under 2-year population, we evaluated whether the lesions that are confidently diagnosed on imaging with AFP correlation could avoid a biopsy. If a policy of biopsying only indeterminate lesions based on imaging and AFP correlation was followed, a biopsy could be avoided in 93% lesions while retaining a high overall accuracy. This can be followed in lesions where the diagnosis is certain or when an upfront resection is possible. Overall, AFP correlation improved the accuracy of CT and decreased the number of indeterminate lesions, but this was not statistically significant. A larger multicentric study would be helpful to clarify these findings.

The strength of our study is that we analyzed data from a single institute with a good sample size of treatment naïve cases for assessing the accuracy. We specifically focused on the under 2-year population, which is seldom researched in isolation. The limitations of our study include a referral bias of being a tertiary oncology center, the retrospective nature of the study, and disproportionately higher cases of hepatoblastomas. We performed a consensus review with two radiologists rather than an independent analysis with interobserver agreement calculation, but we believe that this mirrors the practical scenario more accurately. We also did not evaluate the impact of multidisciplinary team discussions in the cases due to the retrospective nature of the study. Larger prospective studies would be required to evaluate this aspect.

## Conclusion

The overall accuracy of CT with AFP correlation in diagnosing liver lesions in under 2-years age was 92.1% assuming the indeterminate lesions to be inaccurate, and 98.8% if the indeterminate lesions were excluded for biopsy. Presence of calcifications and of a central scar-like appearance was significantly associated with the diagnosis of hepatoblastoma. AFP correlation increased the accuracy of diagnosis by 10.3% and reduced the number of indeterminate liver lesions by 9.1%, although this was not statistically significant. A policy of biopsying only indeterminate lesions after CT and AFP correlation, especially after multidisciplinary tumor board discussion, would avoid sampling in 93% patients.

## Supplementary Information

Below is the link to the electronic supplementary material.

Supplementary file1 (DOCX 255 KB)

## Data Availability

The data analyzed during this study are available from the corresponding author on reasonable request.
