# Supplementary material for: Accuracy of contrast-enhanced CT in liver neoplasms in children under 2 years age
Source: Pediatr Radiol. 2024 Jun 3;54(12):1946–55. doi: 10.1007/s00247-024-05958-w (PMC11579165; doi:10.1007/s00247-024-05958-w)
Supplement: Supplementary file 1 — Supplementary file1 (DOCX 255 KB) [file 247_2024_5958_MOESM1_ESM.docx]

**Supplementary table**

**Table 4.** Definitions of imaging characteristics

| **Imaging characteristics** | **Definition** | **Images** |
| --- | --- | --- |
| Central-scar like appearance | Central hypo-enhancing stellate area within the mass | 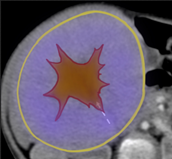 |
| Lobulated morphology | Multiple distinct lobules present within the mass (not to be confused with lobulated margins) | 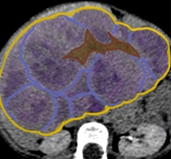 |
| Multilobular arterial enhancement | Patchy multifocal lobular arterial enhancement within the mass, likely arterial enhancement of the various lobules | 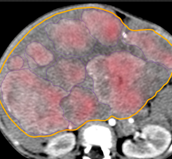 |
| Sieve-like or Swiss-cheese appearance | A predominantly cystic lesion with multiloculatar cysts giving a sieve or Swiss-cheese like appearance | 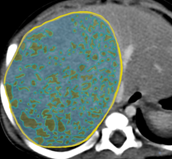 |
